# Benefits of Mobile Phone Technology for Personal Environmental Monitoring

**DOI:** 10.2196/mhealth.5771

**Published:** 2016-11-10

**Authors:** David Donaire-Gonzalez, Antònia Valentín, Audrey de Nazelle, Albert Ambros, Glòria Carrasco-Turigas, Edmund Seto, Michael Jerrett, Mark J Nieuwenhuijsen

**Affiliations:** ^1^ ISGlobal, Centre for Research in Environmental Epidemiology (CREAL) Barcelona Spain; ^2^ Pompeu Fabra University (UPF) Barcelona Spain; ^3^ Ciber on Epidemiology and Public Health (CIBERESP) Barcelona Spain; ^4^ Physical Activity and Sports Sciences Department, Fundació Blanquerna, Ramon Llull University Barcelona Spain; ^5^ Center for Environmental Policy, Imperial College London London United Kingdom; ^6^ Department of Environmental and Occupational Health Services, University of Washington Seattle, WA United States; ^7^ Environmental Health Sciences, School of Public Health, University of California Berkeley, CA United States; ^8^ Department of Environmental Health, Fielding School of Public Health, University of California Los Angeles, CA United States

**Keywords:** smartphone, cell phones, mobile applications, monitoring, ambulatory, spatio-temporal analysis, automatic data processing, travel, environmental exposure

## Abstract

**Background:**

Tracking individuals in environmental epidemiological studies using novel mobile phone technologies can provide valuable information on geolocation and physical activity, which will improve our understanding of environmental exposures.

**Objective:**

The objective of this study was to assess the performance of one of the least expensive mobile phones on the market to track people's travel-activity pattern.

**Methods:**

Adults living and working in Barcelona (72/162 bicycle commuters) carried simultaneously a mobile phone and a Global Positioning System (GPS) tracker and filled in a travel-activity diary (TAD) for 1 week (N=162). The CalFit app for mobile phones was used to log participants’ geographical location and physical activity. The geographical location data were assigned to different microenvironments (home, work or school, in transit, others) with a newly developed spatiotemporal map-matching algorithm. The tracking performance of the mobile phones was compared with that of the GPS trackers using chi-square test and Kruskal-Wallis rank sum test. The minute agreement across all microenvironments between the TAD and the algorithm was compared using the Gwet agreement coefficient (AC1).

**Results:**

The mobile phone acquired locations for 905 (29.2%) more trips reported in travel diaries than the GPS tracker (*P*<.001) and had a median accuracy of 25 m. Subjects spent on average 57.9%, 19.9%, 9.0%, and 13.2% of time at home, work, in transit, and other places, respectively, according to the TAD and 57.5%, 18.8%, 11.6%, and 12.1%, respectively, according to the map-matching algorithm. The overall minute agreement between both methods was high (AC1 .811, 95% CI .810-.812).

**Conclusions:**

The use of mobile phones running the CalFit app provides better information on which microenvironments people spend their time in than previous approaches based only on GPS trackers. The improvements of mobile phone technology in microenvironment determination are because the mobile phones are faster at identifying first locations and capable of getting location in challenging environments thanks to the combination of assisted-GPS technology and network positioning systems. Moreover, collecting location information from mobile phones, which are already carried by individuals, allows monitoring more people with a cheaper and less burdensome method than deploying GPS trackers.

## Introduction

Environmental exposures are crucial determinants of people's health [1]. Despite their importance for health, historically, the assessment of exposure to environmental health risks has been mainly based on exposures derived from the occupational or residential microenvironments [2]. Now, however, it is well known that this approach is inaccurate to represent most of the environmental exposures, given the large spatiotemporal variability of people's activities and microenvironments visited in a day [3-5]. As a result, researchers have started to perform small quasi-experimental studies seeking approaches that move the exposure assessment science from the microenvironment level to the individual level [6-9]. The finding of a reliable and accurate remote tracking tool will provide researchers with the opportunity to determine and understand the causal and temporal relationship of natural and urban environments with health-related behaviors and exposures as well as physical and mental health conditions [10]. However, the previously used tracking tools, such as travel diaries, questionnaires, and Global Positioning System (GPS) technology, along with postprocessing methods have prevented the instauration of this new paradigm of exposure science in epidemiological studies because they are limited and suffer from important weaknesses [11,12].

Mobile phone technology may help to overcome the previous limitations because of its widespread use around the world and the combination of assisted GPS technology and network positioning systems [13-16]. The assisted GPS technology makes use of remote GPS location servers to reduce both power consumption and the time to first fix position [14]. Network positioning systems get geolocation using Wi-Fi signals or, in their absence, from cellular network signals to complement location information from the assisted GPS technology when there is limited satellite visibility. However, until now, mobile phone technology has been assessed only regarding its tracking performance and accuracy mainly in experimental or quasi-experimental studies (ie, scripted studies in a controlled environment and small population sizes) [12,14,17-22]. Moreover, mobile phone technology might not be accurate enough for street-level tracking because of the limitations in GPS antenna, digital interface, GPS chipset, or mobile platform [23].

In this context, the aim of this study was to assess the performance of mobile phone technology in tracking people's travel-activity pattern in a dense city while they perform their daily life activities.

## Methods

### Study Design and Sample

This is a concurrent validation study comparing the tracking and travel-activity determination of a mobile phone versus a GPS tracker and a travel-activity diary (TAD), respectively. The study is nested in the Transportation, Air Pollution and Physical Activities (TAPAS) Travel Survey study [24]. In brief, the TAPAS Travel Survey study aimed to understand the barriers and benefits of bicycle commuting among those who commute by a motorized mode within Barcelona city [25-28].

For this study, a convenience sample of 178 participants from TAPAS Travel Survey study was used. The TAPAS sample was composed of 815 healthy participants recruited following stratified sampling according to commute mode (bicycle vs motorized commuters) in 4 randomized spatiotemporal sampling points, for each of the 10 districts of Barcelona [25]. The TAPAS participants were aged 18-65 years, and they lived and worked or studied in Barcelona city (area: 102 km^2^; population density: 15,687 persons/km^2^). Of these 178 eligible participants, 8 were excluded because of incomplete or imprecise travel diaries, 6 because they did not wear either the mobile phone or the GPS tracker for at least 10 hours on any of the days with TAD information, and 2 because of technical problems with the GPS tracker, leaving 162 participants for analysis.

The study protocol was approved by the Clinical Research Ethical Committee of the Parc de Salut Mar (CEIC-Parc de Salut Mar), and written informed consent was obtained from all participants.

### Instruments and Variables

Participants were instructed to wear a belt with a Samsung Galaxy Y S5360 mobile phone (Samsung Electronics Co Ltd, Suwon, South Korea) and a GlobalSat BT-335 GPS tracker (GlobalSat WorldCom Corp, Taipei, Taiwan) during waking hours for 7 consecutive days and to fill in a TAD for all of their trips throughout the day.

The Samsung Galaxy Y S5360 mobile phone was selected because it has a built-in accelerometer and GPS sensor, it was available in many countries, and it was among the cheapest mobile phones on the market when the study began. It uses Android 2.3.6 and operates with a Broadcom BCM21553 chipset and a BCM4751 GPS module. The Broadcom BCM4751 is a single-chip GPS receiver with 12 channels all-in-view tracking receiver [29]. Its position fix update rate is 1 second. Its accuracy is within 4.8 m for 95% of its measures. Its average reacquisition, warm start, and cold start are done in 1, 30, and 30 seconds, respectively. The mobile phones were equipped with SIM (subscriber identity module) cards with an Internet data allowance of 500 MB/month, an SD card, and an app called CalFit. The CalFit app was selected because it was free, open source, specifically developed for research purpose, and guaranteed the confidentiality of the information collected.

CalFit is a software for Android mobile phones developed by the University of California, Berkeley [30-33], which can be downloaded from the website edmunseto.com [34]. The software uses mobile telecommunications technology (network), assisted GPS, and accelerometer sensors built in the mobile phones to record the time-resolved location and physical activity of participants [6]. The network uses Wi-Fi or cellular network signals to get the geolocation even when there is limited sky visibility. The assisted GPS technology makes use of remote GPS location servers to reduce power consumption and time to first fix position [14]. Each geographical coordinate recorded is differentiated in either the network or assisted GPS according to its origin, and its precision is estimated in meters (ie, accuracy). The accelerometer information is recorded at 10 Hz, in meters per second squared, and is transformed into physical activity intensity in metabolic equivalents (METs) per minute using validated equations [35]. From the physical activity intensity, it is produced the measure of moderate-to-vigorous physical activity duration, which has been shown interchangeable with the ActiGraph accelerometer [35]. For this study, CalFit was set to provide the geographical coordinates and physical activity intensity every 10 seconds.

Once data collection was completed, each geographical coordinate provided by the mobile phone was assigned to 1 of the 4 predefined microenvironments (home, work or school, in transit, and others) using a newly developed spatiotemporal map-matching algorithm. This map-matching algorithm was developed for this study because of the absence of available algorithms for postprocessing the clouds of geographical coordinates generated when participants are at a place. The chosen cutoff points are based on the extensive revision of the mobile phones’ location data. In brief, the algorithm computes the azimuth between sequential coordinates and calculates the circular variance within groups of 30 coordinates in less than 100 m in linear distance. When the circular variance is greater than 0.7, the group of coordinates is identified as a potential place. Then, all coordinates within 30 minutes and 150 m are considered to belong to this spatiotemporal place. Finally, these spatiotemporal places are assigned to a specific microenvironment when distance between the group of coordinates and the geocoded microenvironment is less than 150 m. The rest of the groups of coordinates that do not belong to previous microenvironments are classified as other microenvironments and their central coordinates are calculated.

The GlobalSat BT-335 GPS tracker was selected because of its good performance in the study by Wu and colleagues [12]. This tracker uses the GPS chipset SiRFstarIII and 20 channels all-in-view tracking receiver [36]. Its position fix update rate is variable. Its accuracy is within 10 m for 95% of its measures. Its average reacquisition, warm start, and cold start are done in 0.1, 38, and 42 seconds, respectively. The tracker was calibrated before each deployment following user manual instructions and configured to provide data on date, time, geographical coordinates, speed, bearing, and altitude every 10 seconds. Finally, the TAD used for this study was similar to most travel logs used in transportation studies. It was previously pilot-tested within a convenience sample of 36 participants [6]. It includes questions on start and end time at minute resolution, travel modes, purpose, and destination address for each trip and monitors incidences for each day.

At the end of the study week participants returned the TAD, which was checked, day by day and trip by trip, ensuring that all trips and destinations and their durations and addresses were congruent, helping the participant to correct any illogical situation found. The main travel mode of all multimodal trips of the TAD (n=177 trips) was defined as the most motorized travel mode according to the following ranking: car> motorcycle> bus> metro> bicycle> walk. The geographical coordinates of both mobile phone–based CalFit and the GPS tracker that did not belong to the European continent or with a speed of ≥200 km/h were flagged. Finally, owing to schedule incompatibilities, not all participants were sampled for a week (3 had less than 7 days and 25 more than 7 days). As a result, the total number of monitored days was 1173. Among these, 187 (16%) days were excluded because of the following reasons: (1) the sensors were worn less than 10 hours during waking hours according to the wearing time estimates derived from CalFit physical activity measurements [37]; (2) there was incomplete TAD information; or (3) the participants reported issues with the sensors.

For the analysis, 2 datasets were generated. The first dataset was a trip-level spatiotemporal dataset, with the geographical coordinates of both mobile phone and GPS tracker at 10-second resolution for the episodes identified as trips by the TAD to compare their tracking performance and accuracy. Tracking performance of the trips reported in the TAD was measured by 2 dimensions, identifiability and traceability. Identifiability of TAD trips was defined as having ≥30% of trip duration with geolocation information because it was understood as the minimum cutoff point to distinguish between a real displacement and a measurement error. Traceability of TAD trips was quantified for each identifiable trip by the percentage of the trip duration with geolocation information. On the other hand, the tracking accuracy was quantified by the distance between the geographical coordinates of mobile phone and GPS tracker throughout TAD trips. This distance was calculated between concomitant locations (locations with a difference in time of <10 seconds between both monitors) and corrected for time difference and traveling speed. The second dataset was a microenvironment-level dataset, with information on whether participants were at home, work or school, in transit, or other locations at 1-minute resolution to assess the agreement and variability between map-matching algorithm and TAD.

Finally, other measurements to contextualize participants’ characteristics and built environment around the home included sociodemographic characteristics (eg, age, sex, civil and working status), health status (the question “In general, would you say your health is: Excellent, Very Good, Good, Fair, or Poor” from the SF-36 Health Survey [38]), smoking habits, body mass index, main commute mode, and objectively measured social, physical, and built environment variables of a participant’s residence or neighborhood (eg, deprivation index, population density, distance to work, altitude, slope, and walkability index). Details of these procedures have been previously published [28].

### Statistical Analysis

To assess the validity of the tracking performance of the mobile phone, the identifiability and average traceability of the mobile phone for all trips and for each travel mode were compared with that of the GPS tracker using chi-square test and Kruskal-Wallis rank sum test, respectively. The validity of mobile phone tracking accuracy was assessed by the distance between the concomitant geographical coordinates of the mobile phone and GPS tracker. The tracking accuracy of each mobile phone location was overlapped on a Catalonia street map and a district map of Barcelona city to inspect the spatial coverage and distribution.

On the other hand, the validity of our map-matching algorithm to determine the time in each microenvironment (home, work or school, in transit, and others) was estimated by building a misclassification matrix versus the TAD. From this matrix, the sensitivity (recall), positive predictive value (precision), specificity, negative predictive value, F-score, and Gwet agreement coefficient (AC1) statistics were computed. F-score is the harmonic mean of recall and precision. The AC1 is similar to the multicategory kappa statistic but circumvents the known weakness of kappa [39]. In the AC1 calculation [AC1 = (p0 − pe)/(1 − pe)], “p0” is the concordance observed and “pe” is the concordance expected under the null hypothesis (no relationship).

Finally, two sensitivity analyses were performed. The first one was a comparison between the used geolocation accuracy (based on distance to GPS tracker) and the usual geolocation accuracy (based on distance to nearest street), using only a subset of mobile phone geolocations. The subset includes the geographical locations between the latitudes 41.59 and 41.62 and longitudes 2.605 and 2.645, which belong to the village of Sant Pol de Mar and its surroundings. In the second sensitivity analysis, we assessed the effect of participants' characteristics on the performance of our travel-activity algorithm and the need for specific calibration. The characteristics of participants studied were as follows: (1) main travel mode for commuting; (2) weekdays versus weekend days; (3) median distance from home to work; and (4) working versus studying status. The effect of the characteristics on the performance of the algorithm was assessed by comparing the agreement between abovementioned characteristics using Kruskal-Wallis rank sum test.

All analyses were conducted during 2014-2015, using R 3.1.3 (The R Foundation for Statistical Computing), Python 2.7 (Python Software Foundation), NumPy ≥ 1.6.1 (Travis Oliphant), Pandas ≥ 0.12 (Wes McKinney), SQLite ≥ 3.7.13 (D. Richard Hipp), and SpatiaLite ≥ 4.0.0-RC1 (Alessandro Furieri).

## Results

The 162 participants were on average 33 years old, 50% were female, 20% were single, 40% had at least 1 child, 77% were currently employed, and 50% were bicycle commuters (Table 1). Participants resided in densely populated neighborhoods (mean 30,319 persons/km^2^) and had a relatively short commute distance (mean 3.4 km; Table 1). The total number of valid monitoring days was 986 days (out of 1173 possible days), which represented 3098 trips (mean 19 trips per participant). The average trip frequency was 3 per day, with each trip having an average duration of 28 minutes (Table 2). 85.99% (2633/3098) of these trips were made within Barcelona city (data not shown).

The mobile phone obtained locations for 905 (29%) more TAD trips than the GPS tracker (*P*<.001; Table 2). Their overall traceability, though, was comparable and the median distance between mobile phone and GPS tracker concomitant coordinates (or accuracy) was 24 m overall, 22 m using satellite signal, and 97 m using network signals (from the 1,294,805 compared geographical coordinates). Moreover, the stratified analysis of traceability of trips according to travel mode showed that the mobile phone had better traceability than the GPS tracker, with the exception of car trips.

**Table 1:** Description of participants’ sociodemographic and home characteristics according to the main commute mode.

Characteristics		All Participants (N=162)	Bicycle (n=72)	Car, motorcycle, or bus (n=47)	Underground (n=43)
**Sociodemographic^a^, n (%)**					
	Sex, female	83 (51.2)	34 (47.2)	26 (55.3)	23 (53.5)
	Age in years, median (25th-75th)	33 (26-41)	34 (29-41)	34 (28-42)	27 (21-39)
	Civil status: single	29 (19.3)	17 (25.4)	5 (11.6)	7 (17.1)
	Has at least 1 child: yes	59 (39.3)	22 (32.8)	22 (51.2)	15 (37.5)
	Education level: more than secondary	100 (66.9)	54 (80.6)	25 (58.1)	21 (53.7)
	Working status: yes	115 (76.8)	59 (88.1)	31 (72.1)	25 (63.4)
	Nationality: Spanish	133 (88.7)	59 (88.1)	40 (93.0)	34 (85.4)
	Smoking status: current smoker	42 (28.0)	21 (31.3)	15 (34.9)	6 (15.0)
	Body mass index, ≥25	37 (24.5)	14 (20.9)	15 (34.9)	8 (19.5)	
	High stress level: yes^b^	33 (22.1)	10 (15.2)	12 (27.9)	11 (27.5)
	Health status: very good or excellent	73 (48.3)	35 (52.2)	20 (46.5)	18 (43.9)
**Built environment at home level, mean (SD)**					
	Deprivation index, *z* score	−0.2 (0.7)	−0.3 (0.7)	−0.1 (0.7)	0.0 (0.7)	
	Population density^a^, persons/km^2^	30295 (12212)	32002 (11372)	29575 (12753)	28175 (12859)
	Distance to work, kilometers	3.4 (1.8)	2.8 (1.4)	3.3 (1.8)	4.6 (2.0)
	Slope, %	4.0 (5.3)	3.4 (3.7)	4.5 (6.7)	4.4 (5.8)
	Altitude, meters	41 (42.7)	37 (28.8)	44 (54.2)	44 (48.2)
	Walkability index^a^	0.4 (2.1)	0.6 (2.1)	0.4 (2.1)	0.0 (2.0)

^a^Variables Age, Civil status, Has at least 1 child, Education level, Working status, Nationality, Smoking status, Body mass index and Health status have 12 missing values, High stress level has 14 missing, and Population density and Walkability index have 1 missing.

^b^High stress levels: having a score of ≥4 in each question of the short form of the Perceived Stress Scale [40].

**Table 2:** Comparison of Global Positioning System and mobile phone tracking performance and description of mobile phone tracking accuracy.

Measures		Travel mode from travel-activity diary							
		All	Motorcycle	Walk	Metro	Bicycle	Car	Bus	Others
**Tracking performance**									
Travel diary									
	No. of trips, n	3098	358	706	581	839	409	199	6
	Duration, minutes, mean (SD)	28 (12)	21 (9)	27 (13)	35 (11)	26 (11)	29 (13)	32 (11)	32 (11)
Identifiability^a^									
	GPS^b^ logger, n (%)	1803 (58.2)	280 (78.2)	409 (57.9)	161 (27.7)	574 (68.4)	257 (62.8)	121 (60.8)	1 (16.7)
	Mobile phone, n (%)	2708 (87.4)	311 (86.9)	623 (88.2)	511 (88.0)	766 (91.3)	320 (78.2)	172 (86.4)	5 (83.3)
	*P* value	<.001	<.001	<.001	<.001	<.001	<.001	<.001	.08
Traceability^c^									
	GPS logger, median (25th-75th)	74 (55-88)	80 (65-94)	74 (55-88)	44 (36-53)	76 (61-90)	79 (61-89)	74 (54-87)	86 (86-86)
	Mobile phone, median (25th-75th)	76 (58-90)	77 (60-92)	79 (60-91)	53 (47-62)	85 (71-95)	64 (46-83)	65 (51-84)	87 (87-87)
	*P* value	.009	.60	.009	<.001	<.001	<.001	.05	.32
**Tracking accuracy^d^**									
	Overall, m, median (25th-75th)	24 (10-51)	22 (11-47)	21 (9-46)	22 (11-43)	23 (10-49)	34 (13-76)	25 (12-51)	12 (4-29)
	Satellite, m, median (25th-75th)	22 (10-47)	21 (10-43)	20 (8-43)	21 (10-40)	22 (9-45)	29 (11-61)	23 (11-45)	12 (4-28)
	Network, m, median (25th-75th)	97 (26-574)	66 (22-290)	42 (19-183)	104 (29-609)	69 (23-223)	464 (80-2311)	54 (22-372)	177 (104-242)

^a^Identifiability of travel-activity diary trips was defined as having ≥30% of trip duration with location information.

^b^GPS: Global Positioning System.

^c^Traceability of travel-activity diary trips was quantified among the identifiable trips by the percentage of the trip duration with location information.

^d^Tracking accuracy was quantified by the distance between the geographical coordinates of the mobile phone and the GPS tracker throughout travel-activity diary trips. This distance was calculated between concomitant geographical coordinates (geolocations with a difference in time of <10 seconds between both monitors) and corrected for time difference and traveling speed. Overall includes satellite and network locations, while satellite and network refer to the specific accuracy for each signal.

**Figure 1:**
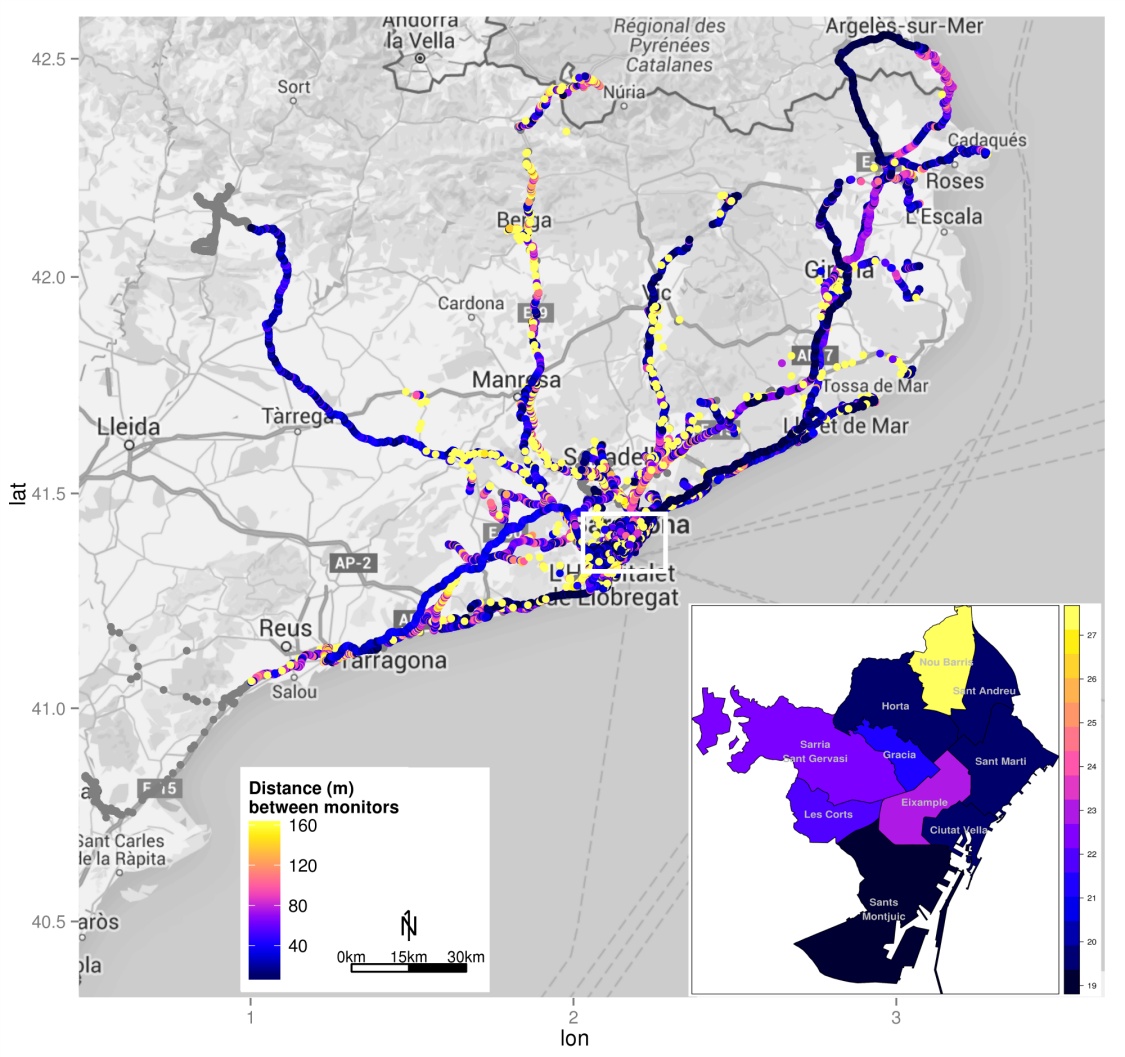
Catalonia street map and Barcelona district map with the spatial distribution of the mobile phone tracking accuracy among the 986 person-days monitored. Gray points represent those locations without concomitant locations from Global Positioning System (GPS) tracker to estimate accuracy. In the district map of Barcelona (inset), the median geolocation accuracy of the mobile phone is shown in the 10 districts of Barcelona city.

**Figure 2:**
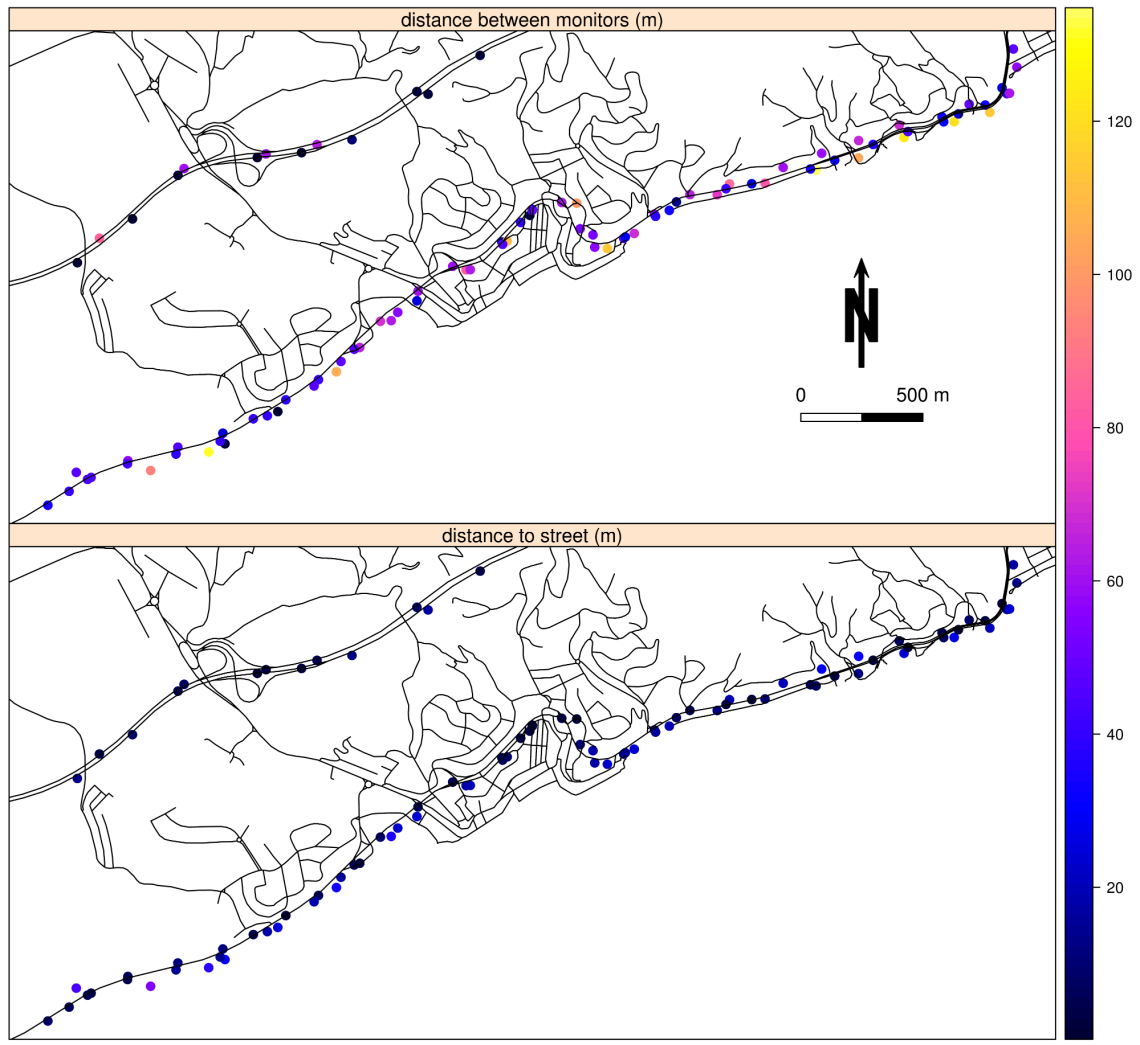
Comparison of distances between smartphone and Global Positioning System (GPS) tracker concomitant locations and to nearest street, while travelling, through the highway C-32 and N-II, at the surroundings of the village of Sant Pol de Mar.

Figure 1 shows the spatial coverage of all sampled trips and how the distance between mobile phone and GPS tracker is greater for the intercity trips. Moreover, the detailed map of Barcelona districts shows that the accuracy of the mobile phone while traveling is almost equal across districts with the exception of Nou Barris district. Figure 2 shows that the distances to the street were less, median 8.7 m (25th-75th, 4.9-17.6 m), compared with the distances between concomitant coordinates, median 46.3 m (25th-75th, 32.7-62.5 m).

The comparison of the overall time in each microenvironment between map-matching algorithm and TAD showed that there is overall a good agreement on time spent in microenvironments, with only 0.1% (work) to 1.2% (other) difference estimated in each type of microenvironment.

The confusion matrix (Table 3) between map-matching algorithm and TAD showed an overall accuracy of 83%. Moreover, the map-matching algorithm in comparison with TAD was able to properly identify the minutes spent at home (recall 94% and precision 93%), at work (recall 85% and precision 90%), in transit (recall 61% and precision 55%), and at other places (recall 61% and precision 64%). Furthermore, sensitivity analyses of the microenvironment agreement between map-matching algorithm and TAD did not show significant differences in any of the subpopulations (data not shown).

**Table 3:** Travel-activity confusion matrix between the travel-activity diary and our map-matching algorithm and its interrelationship statistics (cluster defined as 150 m; Gwet agreement coefficient AC1=81%).

Map-matching algorithm	Travel-activity diary				Sens^a^ %	Spec^b^ %	PPV^c^ %	NPV^d^ %	ACC^e^ %	*F* score %
	Home	Work	Others	Trip						
Home	758495	5235	29575	26580	94	90	93	91	92	93
Work	10320	257200	10110	9140	85	97	90	96	95	87
Others	20575	23000	104315	15485	61	95	64	95	91	62
Trip	20755	15880	27445	79160	61	95	55	96	92	58

^a^Sens: sensitivity.

^b^Spec: specificity.

^c^PPV: positive predictive value.

^d^NPV: negative predictive value.

^e^ACC: Accuracy

## Discussion

### Principal Findings

The main findings of this study are that (1) the mobile phone obtained locations for 905 (29%) more trips than a commercial GPS tracker; (2) mobile phone had enough geolocation accuracy to locate the participants at the street level; and (3) the developed map-matching algorithm was able to determine people's travel-activity pattern with an overall accuracy of 83% and in-transit time with a recall of 61% and precision of 55%.

### Comparison With Previous Studies

#### Tracking Performance and Accuracy of Mobile Phone–Based CalFit

To our knowledge, this is the first study describing and comparing tracking performance and accuracy between a mobile phone and a GPS tracker in free-living conditions. Previous studies were mainly focused on evaluating the geolocation accuracy of mobile phones and were conducted in more car-dependent environments and through experimental designs [12,14,17,18,41]. These previous experimental designs tested the accuracy of the location sensor mainly in favorable environments, such as long unimodal trips, constant speed trips, big and wide streets (eg, Interstate 4 or 5, US Highway 301, or downtown Los Angeles), and used accessories to facilitate signal acquisition (eg, cars with roof carrier to hold mobile phone) [12,18].

In this deployment, the mobile phone–based CalFit obtained locations for 905 (29%) more trips than the GPS tracker. According to previous literature, this could be a consequence of the faster time to first fix position and the use of network positioning systems [12]. On the other hand, the traceability of trips with the mobile phone–based CalFit was almost double the traceability found by Michael and colleagues [17] in their scripted study with the Motorola i760 mobile phone (59% vs 35%). Moreover, in contrast to our results, Michael and colleagues [17] also found that traceability was higher when participants traveled by car, whereas in our study traveling by car was the least traceable travel mode. This difference could be explained because Michael and colleagues had more car measurements in suburban areas, where there are lower barriers for satellite coverage if compared with urban or forest areas. However, we cannot disentangle to what extent these differences could be a result of the placement of the device [42] (in this study, mobile phones were worn on the lower abdomen and the GPS tracker on the left hip), signal problem (lack of Wi-Fi signal during intercity trips), or a real mobile phone technical weakness (such as limitations on GPS antenna, digital interface, or GPS chipset built in the mobile phone).

The geolocation accuracy of mobile phones using only satellite signal in previous dynamic experimental studies was between 2 m and 8 m [12,18], which is more precise than the 22 m found in this study. However, previous studies have assessed only the horizontal error, and because it is known that horizontal error could be different from vertical error depending on satellite distribution [43], differences encountered should be interpreted with caution. Moreover, our results are consistent with Duncan and colleagues' [41] finding regarding the accuracy of GPS trackers in mixed use environments (mean 21 m). On the other hand, the accuracy reached by the mobile phones’ network signal in this study, median 97 m (25th-75th, 26-574 m), is comparable to that found by Zandbergen (74 m) [14]. This could be due to the high density of wireless network access points in Barcelona city (>600 access points only from municipal network) [44]. Furthermore, it is worth noting that the sensitivity analysis on the spatial accuracy (Figure 2) has shown that differences between sensors are more a matter of time alignment or data acquisition rather than spatial error on the coordinates.

#### Travel-Activity Determination Performance

This is the first study to monitor a large sample of adults during a full week while they are performing real-life activities using mobile phone technology. Previous studies mainly focused on commercial GPS trackers and experimental or quasi-experimental designs (Multimedia Appendix 1). Moreover, the built environment conditions of our study area add an extra challenge, when compared with previous study areas (Multimedia Appendix 1), because Barcelona is a smaller city for daily commuting (102 km^2^), has higher population density (15,686 persons/km^2^), and lower prevalence of private motorized transport use (15%). In addition, previous studies did not provide information about travel behavior of participants (frequency, duration, travel mode, or number of other places visited per day). Finally, the definitions of “in-transit” and “other” microenvironments are not consistent across previous studies. Briefly, “in-transit” microenvironments have been defined in two different approaches: a comprehensive approach, which includes all travel modes (car, bus, metro, motorcycle, bicycle, foot, or skate), and a more restrictive approach, which only includes in-vehicle and/or walking modes (the category “others” then includes the rest of the travel modes).

The performance of the map-matching algorithm to determine the time spent at home or work has been shown to be very sensitive and precise, which is consistent with previous research [42,45-47]. Another relevant point in this study is the confirmation that a combined use of the mobile phone–based CalFit and the map-matching algorithm provides a better performance to identify the in-transit microenvironments than previous approaches using only GPS trackers [45,46]. However, our in-transit results, which include all travel modes, are still poor in comparison with those focused on the restrictive definition of in-transit microenvironment (ie, mainly in-vehicle mode) [42,47]. On the other hand, and in agreement with previous literature [41], it is still a challenge to distinguish between places that are very close to each other and to detect very short trips (eg, <10 minutes). Both are very important challenges that need to be addressed carefully by researchers during the study design process because they depend on the urban design of each city and activity pattern of the population.

### Strength and Limitations

The use of the GlobalSat BT-335 as the GPS tracker, which was found by Wu and colleagues [12] to be among the faster devices in terms of time to first fix and with better geolocation accuracy, reinforces the internal validity of our results. Moreover, the extensive and heterogeneous sample of real-life trips, composed by commutes within the very dense city of Barcelona and leisure trips out of the city, shows the robustness of the external validity of our findings. Therefore, and because this study used one of the least expensive mobile phones on the market, in one of the most dense and complex built environments of Europe, one would expect to find similar or even better results with higher range of mobile phones or in less dense cities, which would confirm mobile phones as the reference tool for personal exposure research. Despite the encouraging findings of this study, caution is required until future multicenter studies engaged in different cities replicate these findings with different populations and other mobile phones and settings (ie, other urban design environments or environments with less dense Wi-Fi access points).

The interpretation of the results on tracking performance of TAD trips by mobile phone–based CalFit calls for prudence because this tracking definition is based on the percentage of the trip duration with location information, which does not take into account geolocation accuracy. On the other hand, the present assessment of geolocation accuracy is based on the comparison against a GPS tracker, and it is well known that GPS trackers are affected by environmental factors (ie, visibility and geometry of satellites) [43,48]. So, the distance between mobile phone–based CalFit and GPS tracker may not reflect the lack of geolocation accuracy of mobile phone when the geometry and visibility of satellites were challenging [14,49], but this approach was the most feasible because the 3098 unscripted trips assessed. Furthermore, because of the comparison between the distances to GPS tracker and the distances to nearest street, we know that the lack of accuracy was more a matter of data acquisition than spatial error on the coordinates. On the other hand, the use of the TAD, as a reference value for participants' travel-activity pattern, could have penalized the recall and precision of the map-matching algorithm because of the recall and response biases. Finally, another limitation of this study was not having collected the information regarding the number of satellites in view, the number of satellites used, and the horizontal dilution of precision (HDOP) from the satellite signal in order to improve the recall in the detection of the transitions between microenvironments.

### Applicability and Future Developments

The mobile phone–based CalFit, together with our map-matching algorithm, provides a clean tracking of people's activities, which provides researchers with the opportunity to determine and understand the causal and temporal relationship of natural and urban environments with health-related behaviors and exposures as well as physical and mental health conditions. Moreover, this study is the basis for future studies aiming to assess if this map-matching algorithm of mobile phone geolocation shows the same feasibility and precision in other built environments.

Finally, future improvements in personal monitoring must include making the apps downloadable from the Internet and transferring the measurements through the Internet directly to a cloud server, which we believe will minimize efforts during the deployment and the burden on participants and will increase participants' compliance. Furthermore, future developments should also add automatic algorithms for travel mode recognition and outdoor time determination, probably using additional recorded information from location provider (ie, number of satellites in view, number of satellites used during location determination, and HDOP) and from other mobile phone built-in sensors (ie, barometer and light and sound sensors).

Therefore, the use of mobile phones running the CalFit app provides better information on which microenvironments people spend their time in than previous approaches based only on GPS trackers. The improvements of mobile phone technology in microenvironment determination are because the mobile phones are faster at identifying first locations and capable of getting location in challenging environments thanks to the combination of assisted-GPS technology and network positioning systems. Moreover, collecting location information from mobile phones, which are already carried by individuals, allows monitoring more people with a cheaper and less burdensome method.

## Appendix

Multimedia Appendix 1 Comparison of sample, monitoring duration, setting, travel behavior, and time-activity microenvironments definition across studies focused on time-activity pattern.

## Abbreviations

GPS: Global Positioning System
HDOP: horizontal dilution of precision
TAD: travel-activity diary
TAPAS: Transportation, Air Pollution and Physical Activities
