# Supplementary material for: Benefits of Mobile Phone Technology for Personal Environmental Monitoring
Source: JMIR Mhealth Uhealth. 2016 Nov 10;4(4):e126. doi: 10.2196/mhealth.5771 (PMC5122720; doi:10.2196/mhealth.5771)
Supplement: Multimedia Appendix 1 [file mhealth_v4i4e126_app1.pdf]

Table S1. Comparison of sample, monitoring duration, setting, travel behavior, and time-activity microenvironments definition across studies focused on time-activity pattern.

Author (year)	Sample	Monitoring duration	City	In transit	travel mode	commuting distance and/or duration	Indoor vs Outdoor	Micro-environments	Confusion matrix
**Actual Study**	**162 adults**	**1 week**	**Barcelona, Spain; area: 102.15 km^2; density:15,686.6 hab/km^2**	**walking, biking, bus, metro, motorbike, car, and others**	**YES**	**2.16 h/day**	**NO**	**Home; Work/School; Others; In Transit**	**YES**
Nethery (2014)	54 children	10 day period	Montreal,Canada; area: 365.13 km2; density:4,439 hab/km^2	walking, car, truck, and bus	not specified	≥ 1.30 h/day[b]	YES	Home; In transit; School; Outdoors; Indoors	YES
Wu (2011)	47 adults	3 week days	Wilmington area of the City of Los Angeles [2,273/km2] and the western portion of the City of Long Beach [3,500/km2]	in-vehicle (auto, van, or truck, transit, or other); but outdoors (walking, biking, other)[a]	not specified	1.73 h/ day	YES	Indoor; Outdoor static; Outdoor walking; and in-vehicle travel	YES
Tandom (2003)	45 children	2 preschool days	within a school	not applicable	not applicable	not applicable	YES	in-School; out-School	NO
Elgethum (2007)	31 children	1 non-school weekend day	Seattle, USA; area: 217.2 km2; density: 3,077 hab/km2	all time that is not home, work,school or others.	not specified	1.20 h/weekend day	YES	In-home; In-other; Out-home; Out-other; Transit	YES
Breen (2014)	9 adults	24 hours (7 workdays & 2 non-workdays)	Between central North Carolina [highest density 1425,69 hab/km^2]and EPA campus in Research Triangle Park	in-vehicle (auto, van, or truck, transit, or other)[a]	not specified	> 5 miles; 1.14 h/workdays; 1.82 h/non-workdays	YES	Home-In; Home-Out; Work-In; Work-Out; School-In; School-Out; In-Vehicle; and Other.	YES
Adams (2009)	1 adult	4 workdays	Denver, USA; area: 400 km2 density: 1,561 hab/km2	not specified	not specified	≥ 1.35 h/day[c]	NO	Home; School; and transit	YES
Kin (2012)	1 technician	18 days	Seoul, Korea; area: 605.21 km2 density: 17,000 hab/km2	not specified	not specified	0.90 h/day	YES	Home indoors; Others indoors; Transit; and Walking outdoors	NO

[a] walking, running, biking or others were not included as in transit

[b] Taking only into account those trips longer than 30 minutes.

[c] Average commute in mornings was 29 minute but there is location on 27077 out of 34560 possible observations.
